# Protocol for a randomized controlled trial on risk adapted damage control orthopedic surgery of femur shaft fractures in multiple trauma patients

**DOI:** 10.1186/1745-6215-10-72

**Published:** 2009-08-19

**Authors:** Dieter Rixen, Eva Steinhausen, Stefan Sauerland, Rolf Lefering, Matthias Meier, Marc G Maegele, Bertil Bouillon, Edmund AM Neugebauer

**Affiliations:** 1 Department of Trauma and Orthopedic Surgery, University of Witten-Herdecke at the Hospital Cologne-Merheim, Ostmerheimer Str. 200, 51109 Cologne, Germany; 2 Institute for Research in Operative Medicine, University of Witten-Herdecke, Ostmerheimer Str. 200, 51109 Cologne, Germany; 3 Department for Trauma, Hand and Reconstructive Surgery, University of Cologne, 50937 Cologne, Germany

## Abstract

**Background:**

Fractures of the long bones and femur fractures in particular are common in multiple trauma patients, but the optimal management of femur fractures in these patients is not yet resolved. Although there is a trend towards the concept of "Damage Control Orthopedics" (DCO) in the management of multiple trauma patients with long bone fractures as reflected by a significant increase in primary external fixation of femur fractures, current literature is insufficient. Thus, in the era of "evidence-based medicine", there is the need for a more specific, clarifying trial.

**Methods/Design:**

The trial is designed as a randomized controlled open-label multicenter study. Multiple trauma patients with femur shaft fractures and a calculated probability of death between 20 and 60% will be randomized to either temporary fracture fixation with fixateur externe and defined secondary definitive treatment (DCO) or primary reamed nailing (early total care). The primary objective is to reduce the extent of organ failure as measured by the maximum sepsis-related organ failure assessment (SOFA) score.

**Discussion:**

The Damage Control Study is the first to evaluate the risk adapted damage control orthopedic surgery concept of femur shaft fractures in multiple trauma patients in a randomized controlled design. The trial investigates the differences in clinical outcome of two currently accepted different ways of treating multiple trauma patients with femoral shaft fractures. This study will help to answer the question whether the "early total care" or the „damage control” concept is associated with better outcome.

**Trial registration:**

Current Controlled Trials ISRCTN10321620

## Background

Trauma is a major medical and economical issue of health care systems today and the leading cause of death between the age of 1 and 45 years [1]. The World Health Organization assumes that death caused by accidents, violence and war will even increase until 2020. The implications for the society are serious, as most of the trauma victims are young and at the beginning of their careers [2,3].

Fractures of the long bones and femur fractures in particular are common in multiple trauma patients, but the optimal management of femur fractures in these patients is not yet resolved [4,5]. In principle, there are five methods for the treatment of these fractures: cast, extension, external fracture fixation with fixateur externe, and internal fixation with either intramedullary nail or plate osteosynthesis.

Of the five possibilities of treating femoral fractures, cast or extension therapy was considered standard therapy for long bone fractures in multiply injured patients in the 1960s, but this strategy gradually became obsolete. Observational studies showed that mortality rates were reduced by initial fracture stabilization, independent of the procedure performed (fixateur externe, nail or plate). This led to a new standard therapeutic concept for long bone fracture treatment in multiple trauma patients from the mid 1980s on, termed "early total care" [4,5].

But, the question remained whether primary internal (nail/plate) or external fixation (fixateur externe) is advantageous for this patient population, especially in high risk patients with additional chest or head injuries [4,5]. While nailing (in contrast to plate osteosynthesis) is considered the gold standard for treatment of isolated femur shaft fractures because of its undeniable advantage of weight-bearing structure, it is compromised by the significant distress caused by operation time, blood loss, and insertion of the nail, which may act as a "second hit". Studies comparing reamed and unreamed intramedullary nails show the superiority of the reamed nail [6,7].

On the other hand, advocates of temporary external fixation in multiple trauma patients assert its simplicity with regard to initial treatment, as well as hypothetic advantages regarding patient security with less blood loss and possible reduction in systemic response as well as mortality in specific subgroups of trauma patients, e.g. with additional chest or head injuries. However, hypothetic disadvantages of temporary external fixation must also be considered: planned additional surgery for the secondary definitive procedure with prolonged mechanical ventilation and intensive care unit (ICU) stay, planned additional burden for the patient at secondary surgery, increased infection rates by conversion of external to internal fixation, a certain number of patients in which a conversion will not be possible resulting in impaired functional results, moreover the planned conversion within the first days after trauma may also act as a "second hit" to the patient. The optimal time for conversion from external fixation to a definitive procedure is not clear in patients treated with temporary external fixation and secondary intramedullary nailing [8,9].

With respect to the question of "early total care" or temporary fracture fixation by fixateur externe in *all *multiple trauma patients regardless of their anatomic or physiologic injury severity the literature presents a diversity of studies supporting different views. Both a recently published evidence based guideline [4] as well as a current systematic literature review [5] could neither clarify the optimal time point nor procedure of femoral fracture fixation in multiple trauma patients. In addition, an analysis of the German Trauma Registry including more than 8000 multiple trauma patients showed that management differs widely and depends on both the individual hospital strategy as well as on patient/trauma characteristics [5].

In this respect increasing literature evidence suggests that neither "early total care" nor temporary external fixation with secondary definitive internal osteosynthesis should be considered as standard therapy in *all *patients. Instead, decision making should be dependent on the patient's individual risk by its anatomic, physiologic and immunologic injury severity (risk adapted damage control concept): patients with a low mortality risk can be treated by primary definitive fracture fixation while patients at a higher mortality risk should be treated by damage control in the sense of temporary fracture fixation with a fixateur externe and later secondary definitive treatment when the patients' physiologic and immunological situation has been stabilized on the ICU.

Criteria, which have been recommended by various authors in order to define patients, in whom early definitive osteosynthesis might be disadvantageous are named in table 1[10-20]. Unfortunately, up to date, there is no proof for the superiority of the risk adapted damage control concept with regard to evidence based randomized controlled trials nor are the criteria listed in table 1 validated.

**Table 1:** Criteria, which have been recommended by various authors in order to define patients in whom early definitive osteosynthesis might be disadvantageous: Variables not validated.

**Author (year)^[Reference]^**	**Suggested dependent variable**
Trentz (1978) [10]	Cardiopulmonary, coagulation and metabolic criteria

Sturm (1984) [11]	Thoracic trauma

Seibel (1985) [12]	Oxygen transport, coagulation

Burchardi (1990) [13]	Circulation, coagulation

Van Os (1994) [14]	Cardiovascular condition

Reynolds (1995) [15]	Resuscitation, additional injuries (head, chest, pelvic fracture), delay

Friedl (1996) [16]	ISS

Scalea (2000) [17]	Physiology, additional injuries (head, thorax, abdomen)

Rixen (2001) [24]	Prognostic factors: Age, ISS, GCS, BE, coagulation

Pape (2001) [18]	ISS, additional injuries (thorax, abdomen, pelvis), cardiopulmonary criteria, coagulation, temperature, IL-6, „borderline patients“

Kutscha-Lissberg (2001) [19]	Circulation, volume loss, coagulation

Brundage (2002) [20]	Hemodynamics, preoperative resuscitation

**ISS**: Injury Severity Score; **GCS**: Glasgow Coma Scale; **BE**: Base Excess; **IL-6**: Interleukin 6

Thus, both the evidence-based guideline of the EAST organization [4] as well as a systematic literature analysis [5] concluded that a randomized trial is urgently needed to clarify the question whether primary internal fixation (early total care) or temporary external fixation with internal fixation in a second step (in the sense of risk adapted damage control) should be performed in patients with multiple trauma [4,5].

This trial investigates the differences in clinical outcome of primary internal fixation (early total care) or temporary external fixation (damage control) in multiple trauma patients with femoral shaft fractures. The two procedures will be examined concerning the severity of organ failure [sepsis-related organ failure assessment- (SOFA) score as primary endpoint] [21,22]. Furthermore, it shall be scrutinized to what extent the choice of treatment depends on anatomic and physiologic damage severity.

## Methods/Design

### Trial design

This trial is designed as a randomized controlled two-arm interventional multicenter trial. No blinding is performed. Both damage control and primary definitive surgery are standard care.

### Objectives

Primary objective of the trial is to clarify the question of whether a risk adapted procedure in treating femoral shaft fractures in multiple trauma patients as opposed to an early definitive treatment strategy leads to an improved outcome with respect to morbidity and mortality and if yes at what costs.

Furthermore, it shall be scrutinized to what extent the choice of treatment depends on anatomic and physiologic damage severity.

### Eligibility

#### Inclusion criteria

• Multiple trauma (injury of at least two body regions)

• Injury severity score (ISS) ≥ 16

• Femoral shaft fracture which can be treated in principle by nail or fixateur externe

• Beginning of surgical treatment within 24 hours after trauma

• Patient aged 18 years and older

• Calculated probability of death between 20% and 60% [23,24]

• All factors known which are needed for the calculation of probability of death [age, ISS, Glasgow Coma Scale (GCS), base excess (BE), prothrombin time]

#### Exclusion criteria

• III° open fractures

• Endangerment of the patient by one of both strategies

• Refusal of one of both strategies by either the investigator or the patient

• Start of internal or external fracture fixation before randomization

• Participation in concurrent interventional trials

• Pregnancy

### Reasoning for inclusion and exclusion criteria

The inclusion of patients with multiple trauma and corresponding fractures arises from the question of the study itself. Patients with thoracic or cranial trauma will be included according to systematic review of literature.

As patients might be randomized into the group "primary definitive surgery", patients in whom this procedure is not feasible or can not be performed (begin of surgery not within 24 hours possible or different procedure initiated) must be excluded.

The calculation of the probability of death with the given formula (see below) is a validated method of estimating the probability of death in multiple trauma patients [23-25]. It is essential though that all parameters entered are present at the time of calculation. Patients with missing values that are needed can not be included into the study.

Considering probability of death at randomization will allow an equal distribution of global prognosis in both treatment arms. Because the most important factors that determine prognosis in multiple trauma patients are considered, a comparison of this heterogenic patient collective is possible.

Open fractures are not an exclusion criteria per se [26]. Only III° open fractures will be excluded from this study as many surgeons do not favour the option of nailing in such severe open fractures [27,28].

Patients included in or about to be included in other interventional trials or trials involving drugs of any kind must be excluded out of judicial and scientific reasons.

### Interventions

#### Experimental intervention

Temporary fracture fixation with fixateur externe and secondary reamed intramedullary nailing not earlier than 48 hours after trauma.

#### Control intervention

Primary reamed nailing of the femoral shaft fracture.

Early treatment will be performed, depending on the result of randomization, within 24 h (early) with definitive internal stabilization (control intervention) or in two separate procedures with primary temporary fixation (fixateur externe) and definitive internal stabilization at a later time point (experimental intervention).

The second procedure may not be initiated before 48 hours after trauma unless there are urgent medical reasons demanding early management. These reasons must be documented.

Thus, as soon as the patient stabilizes with ventilation, coagulation, hemodynamics, and the metabolic system secondary surgery can be performed (but not earlier than 48 hours after trauma).

The following criteria are defined as a sign of patient stabilization:

1. Ventilation - paO2/FiO2 > 200 (if ventilated) or no need for ventilation

2. Coagulation - prothrombin time > 60% and platelets > 60,000/μl

3. Hemodynamics - no need for Noradrenalin or Adrenalin and MAP > 60 mmHg

4. Metabolic system - BE >-6.0 mmol/l

Furthermore, none of the signs listed below of systemic or local inflammation should be present:

Temperature > 38.5°C

White blood cell count > 20,000/μl or < 4,000/μl

Local pin infection

### Control/Comparators

The chosen control regimen (primary reamed nailing) is currently as well established in routine care as the experimental intervention (temporary fracture fixation with fixateur externe and secondary reamed nailing not earlier than 48 hours after trauma). As discussed above, so far there is no evidence that one of the treatment regimens is superior to the other [4,5].

### Method of assigning patients to treatment groups

**Figure 1:**
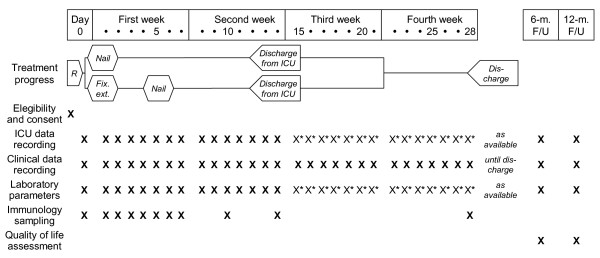
**Frequency and scope of study visits: Simplified visit plan**. R = Recruitment and Randomisation; Fix. ex. = Fixateur externe; F/U = Follow-Up; Data marked with an asterisk (*) will be collected only if they are available from routine measurements.

All multiple trauma patients with femur shaft fractures and age > or = 18 years that present to the involved hospitals will be recorded and eligibility will be checked. The probability of death will be calculated on the trial website using clinical data as described below. If all inclusion criteria are fulfilled the patient will be randomized and documentation will be started (figure 1). Reasons for non-inclusions must be recorded. Allocation concealment will be guaranteed by internet randomization where patients have to be named before allocation to one of the therapy arms. The validated system for internet randomization will be provided by the Coordinating Center for Clinical Studies Cologne (KKSK). This system generates the allocation sequence as follows: The randomisation sequence is blocked using varying block sizes between 6 and 10. Furthermore, randomisation is stratified by study center, in order to adjust for center-specific variation in treatment routines.

### Calculation of probability of death

The probability of death will be calculated according to the following formula of Rixen et al. [23,24]:



With:

BE - Base Excess (mmol/l)

Quick - Prothrombin time (%)

Age - Age of patient

GCS - Glasgow Coma Scale

ISS - Injury Severity Score

This calculation can be done by entering the five variables at the trial website just before randomization. The individual probability of death is visible to the investigator.

### Other baseline characteristics

The pattern and severity of injuries will be documented in detail. For each body region the AIS (abbreviated injury scale) will be used to quantify injury severity. This allows to calculate standard prognostic scores, such as the ISS and the New ISS. In addition the TRISS score will be used to describe injury severity. A fourth more recent score will also be used to describe the severity of trauma. This score, the revised injury severity classification (RISC) score, includes nine variables and has been shown to have very good prognostic capacity. However, as the RISC requires more variables (some of which may not be available in the emergency room quickly), this score will only be used for explorative adjustment of analyses.

In addition to these variables, standard demographic parameters (gender, body mass index, etc.), further physiologic variables (heart rate, temperature, etc.), routine laboratory results on admission (hemoglobin, leukocytes, creatinine, etc.), comorbidities (liver cirrhosis, immunosuppression, renal insufficiency, acute myocardial infarction, etc.) and medical management before randomisation (volume loading in the field and in the emergency room, need for thoracic drainage, radiographs done on admission, etc.) will be recorded.

### Prior and Concomitant Therapy

All concomitant surgical and non-surgical therapy is allowed and key aspects (ventilation, surgery etc.) will be recorded.

### Outcome measures

#### Primary efficacy endpoint

Primary endpoint is the reduction of organ failure as measured by the maximum SOFA score within 28 days after trauma.

The SOFA score has been developed for the sequential evaluation and description of organ failure and has been shown to be a suitable indicator of prognosis in trauma patients [29]. For the present trial, the 5 item SOFA score (exluding CNS) will be used. The central nervous system (CNS) will be excluded due to the known problems in receiving valid data from patients who were intubated and ventilated. Thus the maximum SOFA score will be 20 points (4 points for each organ).

The SOFA score will be assessed daily for the first 28 days after trauma (figure 1). Documentation will start on ICU and will be continued until the patient is returned to a normal ward, where the SOFA score will be set to zero. If the patient is discharged home within the first 28 days, SOFA score is set to zero by definition. If the patient is transferred to another hospital, the last observation will be continued until day 28. Patients who died during the first 28 days after trauma will be assigned the maximum possible SOFA score (20 points) for each day after death.

From the daily assessment of SOFA score, the worst value within the first 28 days will be used as primary endpoint. This corresponds to the intuitive mechanism of reducing the "second hit" which is induced by the operative trauma.

#### Secondary endpoints

• Hospital mortality

• Cumulative organ failure = sum of SOFA score points for the first 28 days

• Incidence of Acute Respiratory Distress Syndrome (ARDS)

• Incidence of Systemic Inflammatory Response Syndrome (SIRS) and sepsis during ICU stay

• Quantity and duration of surgical interventions, anaesthetics, and costs of surgery (material, time)

• Length of ICU stay

• Length of hospital stay

• Number of ventilator-free days

• Therapeutic Intervention Scoring System (TISS) 28 during ICU stay

• Rate of phlegmona of the medullary cavity

• Rate in which conversion to internal fixation was not possible (DCO group only)

• Rate of pseudarthrosis

• Functional outcome at discharge, 6 and 12 months after trauma

• Quality of life at 6 and 12 months after trauma (determined by POLO chart [30])

• Glasgow Outcome Scale (GOS) at discharge, 6 and 12 months after trauma

• Return to work

### Adverse events

All adverse events (AE) during hospital stay will be documented for both study groups. Rate of serious adverse events (SAE), e.g. mortality or ARDS incidence, are part of the secondary endpoints and will be monitored closely. Serious adverse events will be reported to the Principle Coordinating Investigator as soon as possible, at the latest within 24 hours.

Possible complications of the risk-adaptive treatment („damage control orthopedics”, DCO):

Since there are at least two interventions in this treatment arm, there is a hypothetical higher risk of infection in these patients, longer ventilation time, and a certain rate of patients in whom no change from fixateur externe to intramedullary nail is possible and thus, resulting in a higher rate of pseudarthrosis, worse functional result, delayed weight bearing and lower quality of life.

Complications of the primary definitive treatment are primarily the hypothetically higher mortality, increase in duration and rate of SIRS, sepsis and/or multi organ failure (MOF).

Expected Adverse Events

Complications of surgery include:

• Infection

• Delayed healing

• Bleeding

• Nerve injury

• Tendon and muscle injury

• Thrombosis/embolism

• Pseudarthrosis

• Compartment syndrome

### Proposed sample size/Power calculations

The primary hypothesis is that the damage control principle is able to reduce the extent of organ failure. Organ failure is measured daily by the SOFA score (five organs, excluding CNS, 0-4 points per organ). The maximum SOFA score during ICU stay is used as primary outcome measure. It is assumed that the maximum SOFA score could be reduced by 1-2 points. This corresponds to a reduction from "organ failure" to "organ dysfunction", or from "organ dysfunction" to "normal organ function" for one organ. Data from Ferreira et al. indicate that a 2 point increase in SOFA score correlates with an average 10% increase in mortality [29]. According to the database of the Deutsche Interdisziplinäre Vereinigung Intensivmedizin (DIVI; quality assessment in intensive care; 45.000 admissions 2000-2004) maximum SOFA score is about 3.2 ± 3.2 in a general ICU population, and 3.3 ± 3.1 in emergency admissions with trauma. Thus the estimated effect (1.5 points reduction) corresponds to a standardized effect size of 0.5. Assuming usual error rates (a = 0.05; b = 0.20), 64 patients per group would be required. However, due to the non-normal nature of the distribution and the use of non-parametric statistics, the number of patients to be randomized has to be increased by 10%. Thus, the total sample size is set to 140 patients (70 per group). Compliance and rate of loss to follow-up do not affect primary outcome measures.

### Feasibility of recruitment

According to the Trauma Registry of the DGU (1993 - 2004, n = 20.815), 12% of multiple trauma cases had a fracture of the femoral shaft. The limitation to patients with ISS ≥ 16 does not change this rate. A level 1 trauma center treats 50-100 severe trauma patients each year (as observed in the German Trauma Registry). Thus, 15 participating centers will be able to screen at least 1000 patients per year. The number of appropriate patients with femur shaft fractures is 6 - 12 per year per center. Regarding the fact that not every case could be included due to organisational difficulties, only about half of these cases will be included and randomized.

### Statistical analyses

The main outcome variable is the maximum SOFA score during ICU stay. In order to have comparable periods, the time period for SOFA score assessment is limited to the first 28 days after trauma. Documentation of daily SOFA scores is continued in intermediate care or other high dependency units.

The maximum SOFA score will be compared using non-parametric statistics (U-test) due to the substantial skewness of the distribution. Since severity of trauma is assumed to be a major determinant of outcome (including rate of organ failure) the comparability regarding Injury Severity Score (ISS) is tested before analysis. In case of substantial imbalance (p < 0.15; U-test) a stratified analysis of outcome will be performed (two groups; ISS ≤/> 30 points).

Analysis will be done according to intention to treat; regarding to the per protocol (PP) analysis we expect the PP-population to be 5% less.

There will be one interim analysis of data collection when about half of the expected sample size has been included (70 cases). The p-value for the interim analysis will be set at 0.005 so that the final analysis will be done at a significance level of 0.048, according to Fleming and O'Brien [31].

During interim and final analysis, a detailed safety analysis will be performed by comparing predicted and observed survival within each group and reviewing individual cases of non-survivors.

In addition, all secondary endpoints mentioned in the protocol will be considered for explorative analysis, because these factors are repeatedly named in the discussion about the advantages of damage control orthopedics. The aim of presenting all these data is not a confirmative statistical testing, but rather to serve as a basis for comparative analyses in future meta-analyses regarding damage control orthopedics versus early total care strategies.

The hospital perspective on costs includes the initial and all secondary operations or readmissions until one year after randomization. As most costs during hospital stay are caused by surgery itself and ICU care, data collection and statistical analysis will focus on these two aspects. The results will be reported as a cost-effectiveness analysis using life-years lost as the measure of treatment benefit. These analyses will employ decision-analytic models for the optimum management of multiply injured patients with femoral shaft fracture.

### Trial organization

Prof. Dr. med. Dieter Rixen is the Principle Coordinating Investigator.

Participating trial centers and Investigators are listed in the Acknowledgements. Additional trial centres may be added.

#### Trial Steering Committee

The Trial Steering Committee will monitor and supervise the trial and comment on any proposed major protocol amendments (see Acknowledgements).

#### Data safety monitoring board

The data safety monitoring board (DSMB) will ensure the safety of the trial.

#### Trial-supporting facilities

The Coordinating Center for Clinical Trials Cologne (KKSK) will provide the infrastructure for data management and internet randomization.

Statistical analysis will be performed in collaboration with the Institute for Operative Medicine (IFOM) at the University of Witten-Herdecke.

The trial is financially supported by the Deutsche Forschungsgemeinschaft (DFG) [grant number: RI 929/3-1].

### Trial documentation and data collection

Electronic case report forms (eCRF) will be used for documentation. However, quality of life and other self rating parameters are regarded as source data and will only be contained in paper based CRF.

### Quality assurance/Monitoring

In order to guarantee a high quality of the study and data retrieval, all participating centers will be visited on a regular base on site by monitors. Data protection rights will be respected. Randomly selected patients files will be analyzed to control original data and to verify accurate data registration and management (100% source data verification in 15% of the patients). The presence of a written informed consent form and the correct interpretation of inclusion and exclusion criteria will be controlled in all patients. The monitor will particularly concentrate on adverse events, the number of drop-outs, and excluded patients. The investigators in the participating centers will support the monitor in his/her activities.

The steering committee or its designees (clinical monitors) may visit participating centers to control adherence to the protocol. Surgical procedures may be evaluated by the Steering Committee, the DSMB or qualified personnel assigned by it.

The database will be checked for faults and validated by the database programmer. Thereafter the database will be approved for data entry (including full audit trail). Complete and incremental data backup will be performed regularly.

### Ethical consideration

The pros and cons of both treatment principles have been discussed above. Clear evidence for the superiority of one of the strategies does not exist. Nowadays, patients with multiple trauma are treated either with primary external fixation or primary internal nail fixation in the sense of early total care or risk adapted damage control. Which of these two principles will be performed often depends on the general hospital strategy. To give a clear cut picture: which treatment will be applicated depends on the hospital the patient enters by chance, the surgeon on duty by chance, as well as patient characteristics.

Participating centers must be trauma level I centers with great experience in multiple trauma care. Both strategies must be established in the hospital, the operating surgeon must be experienced in both strategies as well. Only patients are enrolled in whom both strategies can be performed and only if the surgeon is capable to perform both strategies in this particular patient. The surgeon is allowed to switch the treatment if there are technical or medical reasons for this.

### Ethics Committee (EC) or Institutional Review Board (IRB)

The final study protocol, including the final version of the written informed consent form, was approved by the ethics committee of the University of Cologne, who is responsible for the Clinical Principal Investigator before commencement of the study.

### Ethical Conduct of the Study

The study is conducted according to ICH-GCP (International Conference on Harmonisation for Good Clinical Practice in clinical research), as set out in the European Union Clinical Trials Directive (2001) and associated UK Regulations (2004), which adhere to the principles of the Helsinki Declaration.

Particular aspects are the study protocol, patient information sheet, informed consent form, submission to EC, administrative documents, data registration, registration of adverse events, preparation for inspection and internal audit by authorized personal or the DSMB, and storage of study documents.

Before inclusion (i. e. before randomization or any other study specific procedure is undertaken), patients will be informed about the trial. However, it is expected that at time of admission, the majority of patients suitable to participate in this trial will not be able to give consent due to the severity of their injuries and/or the nature of prehospital therapy (i.e. intubation). If this situation occurs, informed consent could only be obtained from the patient's legally authorized representative. In the event that the patient's legally authorized representative is not available, the patient can be enrolled under waiver of informed consent. This way of enrollment requires a "Physician Authorization Form", where an independent physician and an impartial witness confirm by signature that all above-mentioned regulations were adhered to. The waiver of informed consent also requires that there is no clue either from the patient's side or from the family members objecting against trial enrollment. The Physician Authorization Form will also be signed by the investigator.

This process of enrollment using the waiver of informed consent is in accordance with German law and international standards of research. Due to the urgency of surgical treatment, it is not possible to obtain informed consent from a legal representative appointed by jurisdiction (i.e. Vormundschaftsgericht). It also is impossible to inform the general public about the trial through public notifications.

The patient will be informed about the trial as soon as possible and will be asked to sign the applicable informed consent form to continue participation in the trial. This consent (or its withdrawal thereof) supersede the authority of any previous authorization for trial enrollment.

Patient information will be updated if new relevant information gets available changing the risk-benefit assessment. Patients already enrolled into the study will be informed by the investigator especially if patient's safety is concerned.

The patient will not pay any costs attributed to the type of procedure or investigational device.

### Publication

Results of the study will be published after approval by the clinical and scientific advisors, Principal Coordinating Investigators, and statistician in an international scientific journal and/or presented on international scientific meetings.

The Principal Investigators will provide the Ethics committee with a summary of the trials outcome, and if applicable the regulatory authorities with any reports required.

All publication will maintain data protection of patient data as well as data of the participating investigators.

### Time Schedule

Inclusion first patient (FPFV): 01.07.2007

Inclusion last patient (Randomization): 01.07.2010

Last patient out (LPLV): 01.07.2011

Database closure: 01.10.2011

Termination statistical analysis: 31.01.2012

Study report: 01.07.2012

## Discussion

Today, there is a trend towards the concept of "Damage Control Orthopedics" in the management of multiple trauma patients with long bone fractures. But, evidence from the current literature is insufficient. As discussed above, results are contradictory, a generalized management strategy is missing. Thus, in the era of "evidence-based medicine", there is the need for a more specific, clarifying randomized controlled trial. Therefore this new trial was initiated.

To our knowledge, this trial is the first to evaluate the risk adapted damage control orthopedic surgery of femur shaft fracutres in multiple trauma patients in a randomized controlled design. In 2003, Pape et al. [32] presented their results of a randomized controlled trial. They investigated the impact of intramedullary instrumentation versus damage control for femoral fractures on immunoinflammatory parameters and complications [32,33]. But, in contrast to this study, where a risk adaption is performed, they excluded multiple trauma patients with severe brain and thoracic injuries (AIS > 3) and patients in unstable or critical condition, too. In summary, the included patients were injured less severely. Furthermore, Pape et al. did not define criteria that must be fulfilled for performing the secondary definitive procedure. Therefore these two trials are not comparable in the authors' opinion.

Since there is still no clear evidence for the superiority of one of the strategies participation in the trial is not associated with an increased risk by itself. If one of the strategies proves superiority the benefit (reducing the organ failure) is evident. The main endpoint is a surrogate endpoint for mortality, i. e. the trial may lead to a definite treatment strategy in patients with multiple trauma lowering the overall mortality. If non-inferiority of the primary internal fixation can be proven, it can be assumed that hospital stay, number of operations etc. are lower in this group, thus lowering the expenses.

Although mortality would have been the most appreciated endpoint, a trial with mortality as the main endpoint would need approximately 1300 patients per arm. In addition to the fact that such a trial is almost undoable for practical reasons the concentration on mortality does not cover all aspects of the planned intervention since the damage control approach primarily tries to limit the sequelae of the "second hit" by surgical intervention. This is reflected by the measurement of organ failure by appointing maximum values for patients who died. The SOFA score has been developed for the sequential evaluation and description of organ failure and has been shown to be a suitable indicator of prognosis in trauma patients [29].

A significant effect may only be shown in a group of medium probability of death (20-60%), especially concerning maximum SOFA-score, as the type of procedure chosen in patient groups of very high or very low mortality will most likely have only a minimal effect on this endpoint.

Because the most important factors that determine prognosis in multiple trauma patients are considered, a comparison of this heterogenic patient collective is possible.

In the current literature, another facet of the optimal surgical treatment in multiple trauma patients is discussed: Biochemical/immunologic factors. It has been shown that the traumatic tissue damage stimulates a maximal activation of the immune system, which leads to its depletion and debilitation [34-36]. As a consequence of a failing immune system a systemic inflammatory response syndrome (SIRS) or sepsis can occur. Both are potentially life threatening complications, in the course of which all organ systems can be affected [34,37,38].

Some of the best analyzed pro-inflammatory cytokines are tumor necrosis factor (TNF)-α [39,40], and the interleukines (IL)-6 and IL-8 [41-43]. Circulating IL-6 is quickly detectable after trauma [42] and has been shown to correlate with trauma load [44].

But so far, the transfer of results into clinical context and the verification for potential decision making (therapy) with respect to risk reduction during secondary surgical procedures after trauma failed.

In extension to the clinical part of the present research a further investigation is planned to monitor both study groups for potential differences in immunofunction/-dysfunction as reflected by temporal patterns of selected circulating mediators and markers for immuno-competence. This approach may result in optimum individualized concepts for fracture repair depending on the patient's individual immuno-competence [45].

Nevertheless, the prerequisites for performing this study give rise to hope for answering the question whether the "early total care" or the „damage control” concept is associated with better outcome.

## Abbreviations

AE: adverse event; AIS: abbreviated injury scale; ARDS: adult respiratory distress syndrome; BE: base excess; CNS: central nervous system; CRF: case report form; CT: computer tomography; DIVI: Deutsche Interdisziplinäre Vereinigung Intensivmedizin; DSMB: data safety monitoring board; DCO: damage control orthopedics; ETC: early total care; GCP: good clinical practice; GCS: Glasgow coma scale; GOS: Glasgow outcome scale; ICU: intensive care unit; ISS: injury severity score; KKSK: coordinating center for clinical studies Cologne; MODS: multi organ dysfunction syndrome; MOF: multi organ failure; OF: organ failure; PI: primary investigator (principle coordinating investigator); RISC: revised injury severity classification; SAE: serious adverse event; SIRS: systemic inflammatory response syndrome; SOFA: sepsis-related organ failure assessment; TISS: therapeutic intervention scoring system; TRISS: Trauma Injury Severity Score.

## Competing interests

The authors declare that they have no competing interests.

## Authors' contributions

DR conceived and designed the trial, drafted the manuscript and secured trial funding. ES prepared the trial's standard operating procedures and eCRF and coordinates multi-centre management. SS contributed to trial design and prepared the trial's standard operating procedures. RL participated in development of the trial protocol and coordinates statistical analyses. MM participated in development of the trial protocol and drafted the manuscript. MGM participated in development of the trial protocol (immunology). BB contributed to trial design. EAMN conceived and designed the trial and secured trial funding. All authors read and approved the final manuscript.

### Participating centers

#### Initiated centers (in alphabetical order)

1. Prof. N. P. Haas, Center for Musculosceletal Surgery, Department of Trauma and Reconstructive Surgery, University of Berlin, Campus Virchow, 13353 Berlin

2. Prof. A. Ekkernkamp, Department of Trauma and Reconstructive Surgery, Unfallkrankenhaus Berlin (UKB), 12687 Berlin

3. Dr. J. Schmidt, Department of Trauma, Hand and Reconstructive Surgery, Helios Hospital Berlin-Buch, 13125 Berlin

4. PD W. Zenker, Department of Trauma and Reconstructive Surgery, Vivantes Hospital Im Friedrichshain, 10249 Berlin

5. Prof. C. Wirtz, Department of Trauma Surgery, University of Bonn, 53127 Bonn

6. Prof. B. Bouillon, Department of Trauma and Orthopedic Surgery, University of Witten-Herdecke at the Hospital Cologne-Merheim, 51109 Cologne

7. Prof. K. E. Rehm, Department of Trauma, Hand and Reconstructive Surgery, University of Cologne, 50937 Cologne

8. Prof. J. Windolf, Department of Trauma and Hand Surgery, University of Düsseldorf, 40225 Düsseldorf

9. Prof. D. Nast-Kolb, Department of Trauma Surgery, University of Essen, 45147 Essen

10. Prof. I. Marzi, Department of Trauma Surgery, University of Frankfurt, 60590 Frankfurt (Main)

11. Prof. R. Hoffmann, Center for Trauma Surgery, Berufsgenossenschaftliche Unfallklinik Frankfurt, 60389 Frankfurt (Main)

12. Dr. K. Brehme, Department of Trauma and Rehabilitation Surgery, University of Halle, 06120 Halle (Saale)

13. Prof. C. Krettek, Department of Trauma Surgery, Medical University Hannover, 30625 Hannover

14. PD F. X. Huber, Department of Trauma Surgery, University of Heidelberg, 69120 Heidelberg

15. Prof. T. Pohlemann, Department of Trauma, Hand and Reconstructive Surgery, Saarland University Hospital, 66424 Homburg/Saar

16. PD M. Wenzl, Department of Trauma, Reconstructive and Hand Surgery, Ingolstadt,

85049 Ingolstadt

17. Prof. A. Seekamp, Department of Trauma Surgery, University Hospitals Schleswig-Holstein, Campus Kiel, 24105 Kiel

18. Prof. C. Josten, Department of Trauma and Reconstructive Surgery, University of Leipzig, 04103 Leipzig

19. Prof. A. Wentzensen, Department of Trauma Surgery, Berufsgenossenschaftliche Unfallklinik Ludwigshafen, 67071 Ludwigshafen

20. Prof. C. Jürgens, Department of Trauma Surgery, University Hospitals Schleswig-Holstein, Campus Lübeck, 23538 Lübeck

21. Dr. R. Ziegelmüller, Department of Trauma Surgery, St. Marien-Hospital Lünen, 44534 Lünen

22. Prof. S. Ruchholtz, Department of Trauma, Reconstructive and Hand Surgery, University Marburg/Giessen, 35043 Marburg

23. Prof. W. Mutschler, Department of Trauma Surgery, University of München, Campus Innenstadt, 80336 Munich

24. PD S. Piltz, Department of Trauma Surgery, University of München, Campus Großhadern, 81377 Munich

25. Prof. M. Nerlich, Department of Trauma Surgery, University of Regensburg, 93053 Regensburg

26. Prof. T. Mittlmeier, Department of Trauma and Reconstructive Surgery, University of Rostock, 18057 Rostock

#### Participating centers, not yet initiated (in alphabetical order)

1. Prof. H.-J. Oestern, Department of Trauma and Reconstructive Surgery, General Hospital Celle, 29233 Celle

2. Prof. K. M. Stürmer, Department of Trauma and Reconstructive Surgery, University of Göttingen, 37075 Göttingen

3. Prof. J. Rueger, Department of Trauma, Hand and Reconstructive Surgery, University of Hamburg, 20246 Hamburg

4. Prof. U. Stöckle, Department of Trauma Surgery, University Hospital Rechts der Isar of the Munich Technical University, 81675 Munich

#### Trial Steering Committee

Prof. Dr. med. Dieter Rixen, Department of Trauma and Orthopedic Surgery, University of Witten-Herdecke at the Hospital Cologne-Merheim, Ostmerheimer Str. 200, 51109 Cologne, Germany

PD Dr. rer. medic. Rolf Lefering, Institute for Research in Operative Medicine, University of Witten-Herdecke, Ostmerheimer Str. 200, 51109 Cologne, Germany

Prof. Dr. med. Marc Maegele, Department of Trauma and Orthopedic Surgery, University of Witten-Herdecke at the Hospital Cologne-Merheim, Ostmerheimer Str. 200, 51109 Cologne, Germany

## References

[B1] Murray CJL, Lopez AD (1997). Alternative projections of mortality and disability by cause 1990-2020: Global Burden of Disease Study. Lancet.

[B2] Lecky F, Woodford M, Yates DW, UK Trauma Audit and Research Network (2000). Trends in trauma care in England and Wales 1989-97. Lancet.

[B3] Scheidt PC, Harel Y, Trumble AC, Jones DH, Overpeck MD, Bijur PE (1995). The epidemiology of nonfatal injuries among US children and youth. Am J Public Health.

[B4] Dunham CM, Bosse MJ, Clancy TV, Cole FJ, Coles MJM, Knuth T, Luchette FA, Ostrum R, Plaisier B, Poka A, Simon RJ (2001). Practice management guidelines for the optimal timing of long-bone fracture stabilization in polytrauma patients: the EAST Practice Management Guidelines Work Group. J Trauma.

[B5] Rixen D, Grass G, Sauerland S, Lefering R, Raum MR, Yücel N, Bouillon B, Neugebauer EAM, AG Polytrauma der DGU (2005). Evaluation of criteria for temporary external fixation in risk-adapted damage control orthopedic surgery of femur shaft fractures in multiple trauma patients: "Evidence-based Medicine" versus "Reality" in the Trauma Registry of the German Trauma Society. J Trauma.

[B6] Bhandari M, Guyatt GH, Tong D, Adili A, Shaughnessy SG (2000). Reamed versus nonreamed intramedullary nailing of lower extremity long bone fractures: a systematic overview and meta-analysis. J Orthop Trauma.

[B7] Rixen D, Sauerland S, Oestern HJ, Bouillon B (2005). Versorgungsstrategien in der ersten operativen Phase nach Verletzung langer Röhrenknochen der unteren Extremität beim Polytrauma: Eine systematische Literaturübersicht. Unfallchirurg.

[B8] Bhandari M, Zlowodzki M, Tornetta P, Schmidt A, Templeman DC (2005). Intramedullary nailing following external fixation in femoral and tibial shaft fractures. J Orthop Trauma.

[B9] Kazakos KJ, Veretras DJ, Tilkeridis K, Galanis VG, Xarchas KC, Dimitrakopoulou A (2006). External fixation of femoral fractures in multiply injured intensive care unit patients. Acta Orthop Belg.

[B10] Trentz O, Oestern HJ, Hempelmann G, Kolbow H, Sturm J, Trentz OA, Tscherne H (1978). Kriterien für die Operabilität von Polytraumatisierten. Unfallheilkd.

[B11] Sturm JA, Oestern HJ, Nerlich ML, Lobenhoffer P (1984). Die primäre Oberschenkelosteosynthese beim Polytrauma: Gefahr oder Gewinn für den Patienten?. Langenbecks Arch Chir Suppl Kongressbd.

[B12] Seibel R, LaDuca J, Hassett JM, Babikian G, Mills B, Border DO, Border JR (1985). Blunt multiple trauma (ISS 36), femur traction, and the pulmonary failure-septic state. Ann Surg.

[B13] Burchardi H, Sydow M, Crozier TA, Burgdorff J (1990). Organversagen bei Polytraumapatienten: Einfluss einer frühen Osteosynthese von Frakturen auf Komplikationen. Anästh Intensivther Notfallmed.

[B14] Van Os JP, Roumen RMH, Schoots FJ, Heystraten FMJ, Goris RJA (1994). Is early osteosynthesis safe in multiple trauma patients with severe thoracic trauma and pulmonary contusion?. J Trauma.

[B15] Reynolds MA, Richardson JD, Spain DA, Seligson D, Wilson MA, Miller FB (1995). Is the timing of fracture fixation important for the patient with multiple trauma?. Ann Surg.

[B16] Friedl HP, Stocker R, Czermak B, Schmal H, Trentz O (1996). Primary fixation and delayed nailing of long bone fractures in severe trauma. Techn Orthop.

[B17] Scalea TM, Boswell SA, Scott JD, Mitchell KA, Kramer ME, Pollak AN (2000). External fixation as a bridge to intramedullary nailing for patients with multiple injuries and with femur fractures: damage control orthopedics. J Trauma.

[B18] Pape HC, van Griensven M, Rice J, Gansslen A, Hildebrand F, Zech S, Winny M, Lichtinghagen R, Krettek C (2001). Major secondary surgery in blunt trauma patients and perioperative cytokine liberation: determination of the clinical relevance of biochemical markers. J Trauma.

[B19] Kutscha-Lissberg F, Hopf FK, Kollig E, Muhr G (2001). How risky is early intramedullary nailing of femoral fractures in polytraumatized patients?. Injury.

[B20] Brundage SI, McGhan R, Jurkovich GJ, Mack CD, Maier RV (2002). Timing of femur fracture fixation: effect on outcome in patients with thoracic and head injuries. J Trauma.

[B21] Vincent JL, de Mendonca A, Cantraine F, Moreno R, Takala J, Suter PM, Sprung CL, Colardyn F, Blecher S (1998). Use of the SOFA score to assess the incidence of organ dysfunction/failure in intensive care units: results of a multicenter, prospective study. Working group on „sepsis-related problems” of the European Society of Intensive Care Medicine. Crit Care Med.

[B22] Vincent JL, Moreno R, Takala J, Willatts S, De Mendonca A, Bruining H, Reinhart CK, Suter PM, Thijs LG (1996). The SOFA (Sepsis-related Organ Failure Assessment) score to describe organ dysfunction/failure. On behalf of the Working Group on Sepsis-related Problems of the European Society of Intensive Care Medicine. Intensive Care Med.

[B23] Rixen D, Raum M, Bouillon B, Lefering R, Neugebauer E, AG Polytrauma der DGU (2001). Base deficit development and its prognostic significance in posttrauma critical illness: an analysis by the trauma registry of the Deutsche Gesellschaft für Unfallchirurgie. Shock.

[B24] Rixen D, Raum M, Bouillon B, Schlosser LE, Neugebauer E, AG Polytrauma der DGU (2001). Prognoseabschätzung des Schwerverletzten - Eine Analyse von 2069 Patienten des Traumaregisters der DGU. Unfallchirurg.

[B25] Matthes G, Seifert J, Bogatzki S, Steinhage K, Ekkernkamp A, Stengel D (2005). Alter und Überlebenswahrscheinlichkeit nach Polytrauma. "Local tailoring" des DGU- Prognosemodells. Unfallchirurg.

[B26] Tornetta P, Bergman M, Watnik N, Berkowitz G, Steuer J (1994). Treatment of grade-IIIb open tibial fractures. A prospective randomised comparison of external fixation and non- reamed locked nailing. J Bone Joint Surg Br.

[B27] Harwood PJ, Giannoudis PV, Probst C, Krettek C, Pape HC (2006). The risk of local infective complications after damage control procedures for femoral shaft fracture. J Orthop Trauma.

[B28] Noumi T, Yokoyama K, Ohtsuka H, Nakamura K, Itoman M (2005). Intramedullary nailing for open fractures of the femoral shaft: evaluation of contributing factors on deep infection and nonunion using multivariate analysis. Injury.

[B29] Ferreira FL, Bota DP, Bross A, Melot C, Vincent JL (2001). Serial evaluation of the SOFA score to predict outcome in critically ill patients. JAMA.

[B30] Pirente N, Bouillon B, Schäfer B, Raum M, Helling HJ, Berger E, Neugebauer E (2002). Systematic development of a scale for determination of health-related quality of life in multiple trauma patients: The Polytrauma Outcome (POLO) Chart. Unfallchirurg.

[B31] Fleming TR, Harrington DP, O'Brien PC (1984). Designs for group sequential tests. Control Clin Trials.

[B32] Pape HC, Grimme K, Griensven M, Scott AH, Giannoudis P, Morley J, Roise O, Ellingsen E, Hildebrand F, Wiese B, Krettek C (2003). Impact of intramedullary instrumentation versus damage control for femoral fractures on immunoinflammatory parameters: Prospective randomized analysis by the EPOFF Study Group. J Trauma.

[B33] Pape HC, Rixen D, Morley J, Husebye E, Mueller M, Dumont C, Gruner A, Oestern HJ, Bayeff-Filoff M, Garving C, Pardini D, Griensven M, Krettek C, Giannoudis P, the EPOFF study group (2007). Impact of the method of initial stabilization for femoral shaft fractures in patients with multiple injuries at risk for complications (Borderline Patients). Ann Surg.

[B34] Bone RC (1996). Toward a theory regarding the pathogenesis of the systemic inflammatory response syndrome: what we do and do not know about cytokine regulation. Crit Care Med.

[B35] Cinat ME, Waxman K, Granger GA, Pearce W, Annas C, Daughters K (1994). Trauma causes sustained elevation of soluble tumor necrosis factor receptors. J Am Coll Surg.

[B36] Cruickshank AM, Fraser WD, Burns HJ, Van Damme J, Shenkin A (1990). Response of serum interleukin-6 in patients undergoing elective surgery of varying severity. Clin Sci (Lond).

[B37] Bochicchio GV, Napolitano LM, Joshi M, Knorr K, Tracy JK, Ilahi O, Scalea TM (2002). Persistent systemic inflammatory response syndrome is predictive of nosocomial infection in trauma. J Trauma.

[B38] Davies MG, Hagen PO (1997). Systemic inflammatory response syndrome. Br J Surg.

[B39] Goodman JC, Robertson CS, Grossman RG, Narayan RK (1990). Elevation of tumor necrosis factor in head injury. J Neuroimmunol.

[B40] Svoboda P, Kantorova I, Ochmann J (1994). Dynamics of interleukin 1, 2, and 6 and tumor necrosis factor alpha in multiple trauma patients. J Trauma.

[B41] Keel M, Ecknauer E, Stocker R, Ungethum U, Steckholzer U, Kenney J, Gallati H, Trentz O, Ertel W (1996). Different pattern of local and systemic release of proinflammatory and anti-inflammatory mediators in severely injured patients with chest trauma. J Trauma.

[B42] Kossmann T, Hans VH, Imhof HG, Stocker R, Grob P, Trentz O, Morganti-Kossmann C (1995). Intrathecal and serum interleukin-6 and the acute-phase response in patients with severe traumatic brain injuries. Shock.

[B43] Pasquale MD, Cipolle MD, Monaco J, Simon N (1996). Early inflammatory response correlates with the severity of injury. Crit Care Med.

[B44] Maegele M, Riess P, Sauerland S, Bouillon B, Hess S, McIntosh TK, Mautes A, Brockmann M, Koebke J, Knifka J, Neugebauer EAM (2005). Characterization of new rat model of experimental combined neurotrauma. Shock.

[B45] Giannoudis PV, Hildebrand F, Pape HC (2004). Inflammatory serum markers in patients with multiple trauma: can they predict outcome?. J Bone Joint Surg Br.

